# Pediatricians’ assessments of caries risk and need for a dental evaluation in preschool aged children

**DOI:** 10.1186/1471-2431-12-49

**Published:** 2012-07-11

**Authors:** C Marshall Long, Rocio B Quinonez, Heather A Beil, Kelly Close, Larry P Myers, William F Vann, R Gary Rozier

**Affiliations:** 1 Department of Pediatric Dentistry, School of Dentistry, University of North Carolina, Chapel Hill, NC, USA; 2 School of Nursing, University of North Carolina, Chapel Hill, NC, USA; 3 North Carolina Department of Health and Human Services, Division of Public Health, Oral Health Section, Raleigh, NC, USA; 4 North Carolina Department of Health and Human Services, Division of Medical Assistance, Raleigh, NC, USA; 5 Department of Health Policy and Management, Gillings School of Global Public Health, University of North Carolina, Chapel Hill, NC, USA

## Abstract

**Background:**

Risk-based prioritization of dental referrals during well-child visits might improve dental access for infants and toddlers. This study identifies pediatrician-assessed risk factors for early childhood caries (ECC) and their association with the need for a dentist’s evaluation.

**Methods:**

A priority oral health risk assessment and referral tool (PORRT) for children < 36 months was developed collaboratively by physicians and dentists and used by 10 pediatricians during well-child visits. PORRT documented behavioral, clinical, and child health risks for ECC. Pediatricians also assessed overall ECC risk on an 11-point scale and determined the need for a dental evaluation. Logistic regression models calculated the odds for evaluation need for each risk factor and according to a 3-level risk classification.

**Results:**

In total 1,288 PORRT forms were completed; 6.8% of children were identified as needing a dentist evaluation. Behavioral risk factors were prevalent but not strong predictors of the need for an evaluation. The child’s overall caries risk was the strongest predictor of the need for an evaluation. Cavitated (OR = 17.5; 95% CI = 8.08, 37.97) and non-cavitated (OR = 6.9; 95% CI = 4.47, 10.82) lesions were the strongest predictors when the caries risk scale was excluded from the analysis. Few patients (6.3%) were classified as high risk, but their probability of needing an evaluation was only 0.36.

**Conclusions:**

Low referral rates for children with disease and prior to disease onset but at elevated risk, indicate interventions are needed to help improve the dental referral rates of physicians.

## Background

Nearly all children have a medical visit by their first birthday, and these outnumber dental visits by 250 to 1 for infants and toddlers [1]. For this reason, the medical home offers an excellent setting to deliver timely preventive oral health care. Accordingly, the American Academy of Pediatrics guidelines recommend that infants receive an oral health risk assessment by their primary care provider prior to age 1 and a dental referral by age 1 when the dental workforce is available [2,3]. These guidelines are important because evidence suggests that physicians’ dental referrals can increase the likelihood and timeliness of a visit to the dentist, particularly among high-risk children with early childhood caries (ECC) [4,5].

The lack of an adequate dental workforce to care for young and vulnerable children underscores potential challenges in a successful physician referral [6,7]. A simulation study of children ages 1-3 years estimated that a referral model whereby pediatricians performed caries risk assessment, referred children at high-risk for ECC to dental providers before the age 3, and continued periodic risk assessments for all other children, decreased untreated ECC. The study also concluded that if all children were referred by age 1 regardless of risk status, high risk, low-income children would be crowded out of the dental care system, effectively diminishing access, particularly under a scenario of a limited dental workforce [8]. These findings suggest that for populations experiencing difficulty with access to dental care, priority risk-based referrals are a key component to more successfully establishing a dental home.

Little is understood about physicians’ referral behaviors and how their assessment of child risk for ECC influences referral patterns. A demonstration project in North Carolina (NC) known as the “*Carolina Dental Home*” (CDH) provided the opportunity to understand how oral health risk factors, as reported by parents and physicians, influence dental referrals. CDH is an extension of Into the Mouth of Babes (IMB), a program initiated in January 2000 to increase access to preventive dental care for NC Medicaid recipients’ younger than 3½ years of age [9]. IMB trains pediatricians, family physicians, and physician extenders to provide dental screenings, oral health counseling, and fluoride varnish applications to children’s teeth. CDH is designed to strengthen the link between medical and dental homes and thus improve referral effectiveness.

Structured checklists are commonly used in primary care to support the dissemination and implementation of clinical practice guidelines because of their demonstrated effectiveness [10]. Well-tested tools for use by physicians to guide oral health risk assessments and referrals generally are unavailable. To provide guidance for physicians in the evaluation of caries risk in infants and young children and their referral recommendations, we developed the Priority Oral Risk Assessment and Referral Tool (PORRT). This checklist-type tool was designed to help physicians identify behavioral, clinical, and health risk factors for ECC and facilitate the determination of the necessity for dental referral. A long-term study is underway to evaluate the impact of PORRT and other strategies on referral practices of physicians and the effectiveness of their referrals.

The purpose of this study is to provide insights into physicians’ judgments of caries risk and their determination of a child’s need for a follow-up evaluation by a dentist based on that child’s risk or clinical status. PORRT forms completed by physicians during the baseline phase of CDH are analyzed to provide these insights. As part of this study, physicians with long-standing experience in providing IMB services received training in the use of PORRT. Relevant statements from the AAP policy (2003) on caries risk assessment and referral were reviewed as well as evidence in support of the behavioral and biological risk factors comprising the PORRT. Clinical training in screening, risk assessment and referral relied on the presentation and discussion of clinical photographs of the normal primary dentition and abnormal dental conditions included in the PORRT.

Participating medical providers had integrated the use of PORRT into their practices at the time of the study, but dentists in the community had not received any special training in infant and toddler oral health. So we consider these findings to provide an assessment of physicians’ determinations of risk and the need for a referral in pediatric patients in communities where comprehensive efforts have not been undertaken to facilitate the linkage between medical and dental homes.

## Methods

### Overall design

The PORRT forms were distributed to 11 medical providers trained to provide IMB services practicing in two contiguous counties in NC. They were completed by physicians during the baseline phase of the CDH initiative (September 2007 – March 2008) at well-child visits for Medicaid recipients’ ages <36 months. Parents provided demographic and behavioral oral health information while the physicians provided clinical information, overall caries risk, and need for evaluation by a dentist. The physicians made the necessary dental referrals and submitted a copy of the PORRT forms for data entry. The study was approved by the UNC-CH Biomedical Institutional Review Board for Research Involving Human Subjects. Approved methods included a full waiver of informed consent and HIPAA authorization.

### Development of PORRT

The PORRT was developed and pilot-tested through a multistep process involving input from practicing physicians and dentists. A review of the literature identified risk factors for ECC in preschool aged children and existing risk assessment tools [11-13]. Available risk assessment tools were reviewed to determine the common risk factors recommended for dental and non-dental providers, the way information was collected, and how overall child risk status was determined. We also relied on our experience with the IMB encounter form used widely in medical offices throughout NC [5,14,15]. The initial PORRT was tested in one large pediatric practice and discussed at a joint meeting of the local medical and dental societies for CDH.

The resulting PORRT form documents 11 risk factors for ECC in three domains: behavioral and environmental factors (oral hygiene, diet, sleeping practices, fluoride use, and family dental problems), clinical factors (visible plaque, non-carious enamel defects, non-cavitated [white spot] carious lesions, cavitated carious lesions) and special health care needs. Pediatricians also were asked to provide an overall assessment of ECC risk using an 11-point scale (0 = extremely low risk to 10 = extremely high risk), and to determine if a dentist evaluation was needed (yes, no, don’t know).

### Variable construction

A child’s dental caries risk was measured in three ways. First, risk factors were considered independently and coded as a binary variable with presence of the risk factor as “1” and absence as “0”. Second, scores on the overall risk scale were coded as high risk (6-10) versus other (0-5) based on the frequency score distribution. Third, we classified children into risk categories (low, moderate, high) according to their number and type of risk factors. “High” risk included children with cavitated lesions and/or those identified as having special health care needs. “Moderate” risk included children without special healthcare needs or cavitated lesions, but with three or more risk factors, white spot lesions, or enamel defects. “Low” risk children were those with fewer than three risk factors, and no clinical signs of disease or special health care needs. These risk classification levels reflected consensus among providers participating in the study regarding children younger than 3 years of age who should receive preventive dental care in the medical office and those needing a priority referral to a dentist.

The outcome variable was constructed as a binary variable with a ‘yes’ to the question “Does this child need to be evaluated by a dentist as a result of this assessment?” coded “1” and other responses as “0”. This variable measured physicians’ opinions about the need for a follow-up assessment by a dentist rather than the recommendation that they actually made or would make after considering parent demand factors or the supply of dentists in their community. Our approach therefore helps eliminate patient demand and workforce issues that might affect referral and provides an indicator of their opinions controlling for these two external factors.

### Statistical analysis

A descriptive analysis examined the percent of patients with each risk factor. We tested whether each risk factor was associated with the child’s need for a dental evaluation using a Fisher’s exact test and by calculating unadjusted odds ratios.

Two multivariate logistic regression models examined the independent association between each risk and the need for dental evaluation. One model excluded the caries risk scale because of its strong relationship with the outcome and desire to determine the association between each risk factor and need for dental evaluation without the potential of obscuring these associations. The pediatricians’ overall assessments of risk using the scale also is likely to be based on many of the individual risk factors and can be highly correlated with them. Of the two variables related to brushing, we included one measuring use of fluoridated toothpaste because of its greater importance in caries prevention and potential to increase efficiency of estimates by using a smaller number of variables in the models.

To minimize potential bias from missing data, multiple imputations of the outcome variable (need for dentist evaluation) and each of the included individual risk factors were performed to impute values for missing data [16]. Using the Markov Chain Monte Carlo method from the SAS MI procedure 20 datasets were imputed. We used observations created from the 20 datasets to analyze the effect of each risk factor on the likelihood of the need for a dentist evaluation with logistic regression models.

A third logistic regression model determined the log odds of the physician-determined need for a dentist evaluation based on the three levels of risk. We used the resulting beta coefficients from the logistic regression model to calculate the predicted probability of referral to the dentist based on risk status. All tests used p < 0.05 and SAS Version 9.1 for all analyses [17].

## Results

### Descriptive results

A total of 1,288 PORRT forms were completed. Behavioral risk factors related to sugar and fluoride exposures were highly prevalent. (Table 1) Visible plaque was the most prevalent (8.7%) of the clinical factors, followed by non-cavitated lesions (7.0%), enamel defects (4.5%), cavitated lesions (1.9%), and other oral conditions (1.5%). The caries risk assessment scale had a mean of 3.35/ person (SD = 1.82). Among the 1,232 children in the sample with non-missing individual risk factor information, 78 (6.3%) were classified as high, 505 (41.0%) as moderate, and 649 (52.7%) as low risk.

**Table 1:** Percent of children with ECC risk factors and need for evaluation by a dentist

Risk Factor	Total N	Overall % with risk factor	N with non-missing referral data	Non-missing % with risk factor	% Evaluated as needing a referral by whether or not child has risk factor		Unadjusted Odds Ratio
					Has risk factor	Does not have risk factor	
**Behavioral Risk Factors**							
Teeth not brushed at bedtime	1278	17.92	911	18.22	3.61	6.98	0.50
Teeth not brushed with fluoride	1270	60.00	911	61.03	4.50	9.86	0.43**
Do not drink tap water with fluoride	1267	48.86	904	51.66	6.00	6.86	0.87
Sugary beverages between meals	1271	67.98	904	65.15	8.32	3.17	2.77**
Family dental problems	1270	17.56	902	16.52	11.41	5.31	2.30**
Sleeps with a bottle	1267	16.81	901	15.32	5.80	6.68	0.86
**Clinical Risk Factors**							
Cavitated lesions present	1208	1.90	910	2.09	63.16	5.27	30.78**
Non-cavitated lesions present	1206	7.05	909	6.49	37.29	4.35	13.07**
Enamel defects present	1206	4.56	909	4.51	46.34	4.72	17.42**
Visible plaque present	1206	8.79	909	8.91	29.63	4.11	9.83**
Other oral conditions	1200	1.50	905	1.66	73.33	5.28	49.32**
**Child Health**							
Special Health Care Need	1097	5.20	832	3.97	18.18	5.38	3.91**
**Overall risk scale score**							
High score (> 6)	1133	7.59	877	5.82	52.94	3.63	29.85**

* Statistically different at the p < .05 level; ** statistically different at the p < .01 level, children needing to see the dentist different from other children using univariate logistic regression.

Information regarding the need for a dental evaluation was provided for 917 (71%) of the 1,288 PORRT forms. Of these, only 60 patients (6.5%) were identified as needing a dentist evaluation at the time of the PORRT completion.

### Association of individual risk factors with need for evaluation

Most individual risk factors were associated at a statistically significant level (P < 0.05) with the need for a follow-up evaluation by a dentist (Table 1). All clinical risk factors were strongly associated with need for follow-up, with behavioral risk factors being less so. Individual behavioral risk factors predictive of the need for a dental evaluation included lack of bedtime brushing, lack of fluoridated toothpaste use and family history of dental problems. Odds ratios for the overall risk scale (unadjusted OR = 29.8) and cavitated lesions (unadjusted OR = 30.7) were similar, but second to other oral conditions (unadjusted OR = 49.3) in the strength of their association with the outcome variable.

Results of the regression model including the caries risk assessment scale (high = 6-10 vs. other = 0-5) showed it to be the strongest predictor for a dental evaluation (OR = 10.45, 95% CI =7.34, 14.90) when all risk factors were considered simultaneously (Table 2). All clinical variables and special health care needs remained statistically significant in the regression model that omitted the overall risk scale, but associations between clinical risk factors and need for an evaluation were stronger as measured by the increase in the odds ratios. The presence of cavitated (OR = 17.5, 95% CI = 8.08, 37.97) and non-cavitated lesions (OR = 6.9, 95% CI = 4.47, 10.82) was strongly associated with the need for dental evaluation. The only behavioral risk factor significant in this regression model was a history of family dental problems.

**Table 2:** Association of individual risk factors with need for dental evaluation

Risk Factor	Model 1: Risk scale included		Model 2: Risk scale not included	
	Odds Ratio	95% Confidence Interval	Odds Ratio	95% Confidence Interval
*Behavioral risk factors*				
Does not brush with fluoride	0.83	0.69 - 1.01	0.83	0.67 - 1.04
Does not drink tap water	0.98	0.80 - 1.20	0.99	0.82 - 1.20
Sugary beverages between meals	0.97	0.78 - 1.20	1.05	0.83 - 1.33
Family dental problems	1.22	0.96 - 1.56	1.31*	1.02 - 1.67
Sleeps with a bottle	0.84	0.65 - 1.10	0.94	0.71 - 1.23
*Clinical risk factors*				
Cavitated lesions present	7.01**	2.93 - 16.73	17.52**	8.08 - 37.97
Non-cavitated lesions present	3.11**	1.87 - 5.19	6.95**	4.47 - 10.82
Enamel defects present	1.20	0.60 - 2.40	2.51**	1.44 - 4.35
Visible plaque present	2.56**	1.81 - 3.61	2.95**	2.12 - 4.10
*Child health*				
Special health care needs	2.19**	1.31 - 3.66	2.54**	1.37 - 4.69
*Overall risk scale score*				
High risk on scale	10.45**	7.34 - 14.90	-	-

N = 1,288 with multiple imputation for missing values.

*Statistically different at the p < .05 level ** Statistically different at the p < .01 level.

### Association of risk classifications with need for evaluation

**Figure 1:**
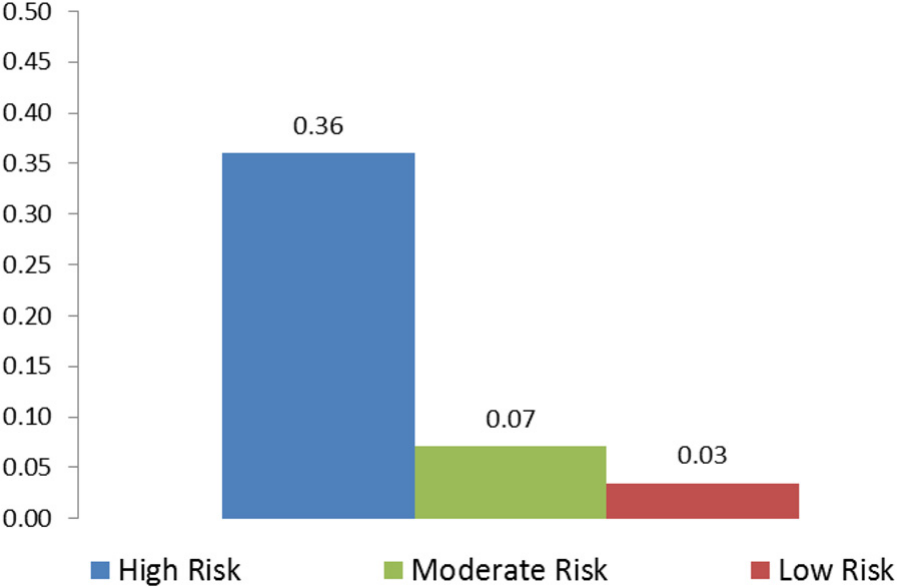
Probability of need for dental evaluation according to risk status.

Figure 1 illustrates the probabilities of needing a dental evaluation according to the three risk categories. The probability of a child being identified as needing a dental evaluation among those considered high risk was only 0.36, but these children had a 15.9 (95% CI = 7.34, 34.41) times higher odds of being identified as such than the low risk group. The probability of needing an evaluation for those children classified as having moderate risk was 0.07, but they had 2.1 (95% CI = 1.11, 4.17) the log odds when compared to the low risk group (results not shown). Finally, the probability for evaluation among those children considered low risk by physicians was only 0.03.

## Discussion

The findings from this study provide useful insights into the oral health risk assessment practices of physicians, the prevalence of risk factors found in this age group of children, and the effects of these factors on physicians’ opinions about the need for a dental referral. These findings are particularly relevant to understanding risk assessment and referral practices of physicians who have been trained to provide oral health services. Participants in this study had received training in risk assessment, counseling and fluoride therapies, but had not participated in any training or community-wide interventions to facilitate dental referrals by physicians.

We found that behavioral risk factors were much more prevalent in this very young patient population than clinical risk factors or dental disease. Three of the six behavioral risk factors were highly prevalent. Limited fluoride exposures and over exposure to sweetened beverages were widespread, with physicians reporting that nearly 50% of their patients had these risks. These findings emphasize the importance of early intervention to reduce the risk of subsequent disease.

Although some of the individual behavioral risk factors were highly prevalent, they had little influence on physicians’ opinions about the need for a dental evaluation. The reasons for this finding could not be explored using information available in this study. However, anticipatory guidance is a major part of well-child visits, with both parents of young children and providers reporting high rates of anticipatory guidance for traditional topics [3]. For example, more than 70% of parents of infants and toddlers report discussing food and feeding issues during health supervision visits [18]. Some of these topics target behaviors that are common risk factors for both dental and medical problems. Because pediatric providers are experienced in providing anticipatory guidance for many behaviors, it is possible that they are confident in providing counseling to address behavioral risk factors for oral health, particularly consumption of sweetened beverages, the most common of the behavioral risk factors found in this study and a primary target for obesity counseling. Because counseling for some of these common risks is so much a part of their usual practice, physicians might not see the need to refer patients to dentists for similar services, particularly when parents will be faced with many difficult challenges in finding a dentist. Primary care physicians might consider their relationship with dentists similar to that of other health specialists to whom they refer mostly when the child has a medical condition that they are unable to treat in their own office.

A history of family dental disease was the only behavioral risk factor independently associated with need for dental evaluation in either of the regression models. The existing literature offers some support for the importance of this finding as there are many parental characteristics associated with increased risk for ECC. [19-21] For example, maternal untreated caries is reported to nearly double the odds of their children having untreated dental disease and increased risk of disease severity [22]. Evidence on the significance of caregiver transmissibility of oral flora to the child is well documented, with limited evidence, however, that caregiver reduction of *Streptococcus mutans* subsequently reduces ECC [23]. It is likely that family dental disease as a variable captures other environmental risks for dental disease that are common for all family members.

All clinical conditions included in this study were strongly associated with the need for dental evaluation. Dental caries was the strongest predictor, with the presence of cavitated (OR = 17.5; 95% CI = 8.08, 37.97) and non-cavitated (OR = 6.9, 95% CI = 4.47, 10.82) lesions greatly increasing the odds of a needed evaluation by a dentist. A previous survey of pediatric primary care providers found that physicians are more likely to refer young children if they have untreated dental caries (85% early disease; 98% extensive disease among those who refer) than if they have a low probability of disease (25.6%) [24]. While early identification and referral of those with disease is important, another goal of screening and risk assessment is to refer children at high risk prior to the onset of disease so that services can be provided to prevent the development of ECC [2].

Considering that the consensus recommendation for professional dental organizations is the universal age 1 dental visit, the large number of risk factors present in this population, and the published literature on the rate of dental referrals by primary care providers, the opinions of physicians about the need for dental evaluation (6.8%) appears to be less than optimal. A national study found that 44.6% of low-income parents of children 2-5 years of age were advised by a non-dental provider to schedule a dental check-up [4]. A study of dental referrals by physicians participating in IMB found an overall referral rate of 2.8%, lower than the one found in this study [5].

The literature reveals a number of factors that can help explain the low dental referral rates for infants and toddlers [24]. Among these are physicians’ confidence in identifying the need for a referral, the availability of dentists, and a practice with a high patient volume of young children. The low rate of referral for children in this study who were observed by physicians to have elevated risk status because of harmful behaviors but no disease adds the possibility of another potential barrier to referral. Physicians might not refer for services done in the dental office that they commonly provide in their own offices. Further study is needed to better understand why referral practices of physicians do not adhere to recommended guidelines, but in the interim, interventions can be undertaken to encourage dental referrals in geographic areas having an adequate dental workforce.

Physicians’ determinations of a child’s overall caries risk as recorded on the risk scale included in the PORRT was a stronger predictor of a needed dental evaluation than any of the individual risk factors in the regression analysis. Those children judged to be at high risk on the scale had a 10-fold greater likelihood of requiring a dental evaluation compared to those judged to be at other risk levels. Although caries risk assessment is used commonly in clinical practice, our use of the overall risk scale has specific methodological considerations. The risk assessment scale was scored after all individual risk factors were recorded on the structured form, preventing providers from scoring the scale without influence from the list of risk factors. So we do not know how the scale would perform if used alone. Use of an overall risk assessment scale to determine priority referral status also has practical disadvantages. An assessment, identification, and record of individual risk factors is useful in selecting counseling strategies and content during a visit, and for monitoring behaviors over time.

Our classification of children according to the number and type of risk factors provides some insights into providers’ possible perceived urgency of a referral. Children with obvious disease (cavitation from caries) or with special healthcare needs should have an immediate referral. Those with early stage disease or a significant number of risk factors can be considered to have a high degree of referral urgency. Those with only a few modifiable risk factors but no disease or special health care need should be referred if the supply of dentists is adequate, but the urgency of that referral can be consider low in young children. The predicted probability of pediatricians’ indicating the need for dentist evaluation for patients with risk factors that would require an immediate referral was only 0.36. This finding is consistent with the study by Pahel and colleagues [5] where referral rates for children with obvious dental disease was only 33%, leaving an opportunity for improvement in the timely establishment of a dental home for those children in need of treatment.

Results of this study need to be considered in the context of its limitations. The presence of clinical risk factors and caries risk assessments were performed by pediatricians without an independent validation of the children’s oral health condition. So the accuracy of their risk assessments and the need for follow-up by a dentist are unknown. Furthermore, like other oral health risk assessment tools for oral health, PORRT has not been tested for its reliability and validity. Age and other potentially important covariates related to risk for dental disease and outcomes were not available, but all children included in the study were enrolled in Medicaid and were younger than 3 years of age. The study population thus represents a fairly homogenous group of children.

A final consideration is that our primary explanatory variable assessed the provider’s opinion about the need for dental evaluation. We do not know to what extent this opinion would result in an actual recommendation for a referral. With a number of factors ultimately influencing whether a referral is made or not, the effectiveness of PORRT in helping to improve access to dental care remains unclear. However, this initial experience with PORRT has provided evidence that physicians are willing to use structured forms to help identify ECC risk factors and the need for an oral evaluation by a dentist.

## Conclusions

1. Pediatricians’ perception of overall caries risk of a child, as recorded on a 11-point risk scale, is a stronger predictor of need for an evaluation by a dentist than individual behaviors, clinical risk factors or actual untreated dental caries.

2. A history of dental disease in the family is the only independent behavioral risk factor predicting need for an evaluation in either of the regression models, possibly capturing both biological and behavioral markers for caries risk in children.

3. Dental disease is more strongly associated with need for referral than behavioral risk factors.

4. Low referral rates for children with disease and prior to disease onset suggest that interventions are needed to help increase guideline adherence and improve dental referral rates for physicians.

## Abbreviations

ECC, Early Childhood Caries; PORRT, Priority Oral Risk assessment and Referral Tool; CDH, Carolina Dental Home; IMB, Into the Mouths of Babes; OR, Odds Ratio; CI, Confidence Interval.

## Competing interests

The author(s) declare that they have no competing interests.

## Authors’ contributions

CML and RQ participated in data analysis and drafting the manuscript. LM, KC and BV helped design and coordinate the study. GR helped design the study, participated in data analysis, and drafting the manuscript. HB performed the statistical analysis and helped draft the manuscript. All authors read and approved the final manuscript.

## Pre-publication history

The pre-publication history for this paper can be accessed here:

http://www.biomedcentral.com/1471-2431/12/49/prepub
